# Solitude and Loneliness Across the Lifespan

**DOI:** 10.1093/geroni/igaf122.366

**Published:** 2025-12-31

**Authors:** Gizem Hueluer, Jana Nikitin

## Abstract

Solitude and loneliness are complex, multifaceted experiences that shape individuals’ well-being across the lifespan. This symposium presents novel perspectives on how solitude and loneliness are shaped by media use, personal motivations, age related factors, and life transitions. Dwight Tse examines social integration and loneliness among older digital gamers, comparing them to non-gamers in a large-scale secondary data analysis of the UK Biobank. Gizem Hueluer presents findings from two experience sampling studies on media use during solitude, exploring how different forms of media consumption are related to loneliness. Jana Nikitin investigates situational solitude in an experimental study, framing it as an opportunity to pursue intrinsically motivated goals and examining its potential to reduce loneliness. Jennifer Lay examines age differences in the recall of solitude experiences. Iris Wahring analyzes loneliness during relationship transitions, conceptualizing solitude as a life situation. Together, these presentations provide key insights into how solitude intersects with media use, motivation, age-related factors, and life transitions in shaping loneliness, contributing to a deeper understanding of these interconnected phenomena across the lifespan.

